# Impact of Noradrenaline Administration Dosage on the Occurrence of Peripheral Intravenous Catheter–Related Venous Phlebitis in Critically Ill Patients Using a Time-Dependent Multilevel Cox Regression Model

**DOI:** 10.1155/emmi/4457109

**Published:** 2025-05-06

**Authors:** Hideto Yasuda, Claire M. Rickard, Jessica A. Schults, Nicole Marsh, Masahiro Kashiura, Yuki Kishihara, Yutaro Shinzato, Shunsuke Amagasa, Takashi Moriya, Yuki Kotani, Natsuki Kondo, Kosuke Sekine, Nobuaki Shime, Keita Morikane, Takayuki Abe, AMOR-VENUS Study Group

**Affiliations:** ^1^Department of Clinical Research Education and Training Unit, Keio University Hospital Clinical and Translational Research Center (CTR), Shinanomachi 35, Shinjuku-ku, Tokyo 160-8582, Japan; ^2^Department of Emergency and Critical Care Medicine, Jichi Medical University Saitama Medical Center, 1-847, Amanuma-cho, Omiya-ku, Saitama-shi, Saitama 330-8503, Japan; ^3^School of Nursing, Midwifery and Social Work, UQ Centre for Clinical Research, The University of Queensland, Building 71/918, Royal Brisbane & Women's Hospital Campus, Herston 4029, Queensland, Australia; ^4^School of Nursing and Midwifery, Alliance for Vascular Access Teaching and Research, Griffith University, Nathan 4111, Queensland, Australia; ^5^Herston Infectious Diseases Institute, Nursing and Midwifery Research Centre, Royal Brisbane and Women's Hospital, Metro North Health, Herston, Queensland, Australia; ^6^Department of Intensive Care Medicine, Kameda Medical Center, 929 Higashi-cho, Chiba, Kamogawa 296-8602, Japan; ^7^Department of Emergency Medicine, Koga Community Hospital, 2-30-1 Daikakuji, Shizuoka, Yaizu 425-0088, Japan; ^8^Department of Medical Engineer, Kameda Medical Center, 929 Higashi-cho, Chiba, Kamogawa 296-8602, Japan; ^9^Department of Emergency and Critical Care Medicine, Graduate School of Biomedical and Health Sciences, Hiroshima University, 1-2-3 Kasumi, Minami-ku, Hiroshima 734-8553, Japan; ^10^Division of Clinical Laboratory and Infection Control, Yamagata University Hospital, 2-2-2 Iidanishi, Yamagata-shi, Yamagata 990-9585, Japan; ^11^Biostatistics, Clinical and Translational Research Center, Keio University School of Medicine, Shinanomachi 35, Shinjuku-ku, Tokyo 160-8582, Japan; ^12^School of Data Science, Yokohama City University, 3-3-1 Ushikubo-Nishi, Tsuzuki-Ku, Kanagawa, Yokohama 224-8551, Japan

**Keywords:** catheterization, noradrenaline, peripheral, phlebitis, spline curve analysis, time-dependent factor

## Abstract

**Purpose:** Peripheral intravenous catheter (PIVC)–administered noradrenaline offers faster treatment for septic shock but risks complications like phlebitis. We aimed to investigate the relationship between the total noradrenaline dose administered via PIVCs and the development of phlebitis by considering the influence of noradrenaline as a time-dependent covariate.

**Methods:** A post hoc analysis was conducted on prospective multicenter cohort data from 23 intensive care units in Japan. The total noradrenaline dose was included as a time-dependent variable in a multilevel Cox regression model, and smoothing splines assessed nonlinear relationships. The primary endpoint was phlebitis. Directed acyclic graphs were used to define confounding factors for the analysis.

**Results:** The analysis included 3410 PIVCs from 1351 patients, with noradrenaline administered to 70 patients (5.2%) with 91 PIVCs (2.6%). The median dwell time and interquartile range of PIVCs was 46.2 h (21.3–82.9). No significant association was observed between the total noradrenaline dose and the occurrence of phlebitis through analysis using the multilevel Cox regression model with time-dependent covariate, which assumed the linear relationship between phlebitis occurrence and the total noradrenaline dose (hazard ratio 1.06, 95% confidence interval [CI] 0.93–1.20). Spline curve analysis suggested a nonlinear relationship between the total noradrenaline dose and phlebitis, and the risk of phlebitis increased when the total administered dose of noradrenaline exceeded 6 mg as the lower limit of the 95% CI exceeded the significant threshold of 1.0. Sensitivity analyses, including additional potential risk factors, showed consistent results compared with those of the primary analysis.

**Conclusions:** Administering noradrenaline within a total dose not exceeding 6 mg reduces the risk of phlebitis, potentially allowing safer administration through PIVCs.

**Trial Registration:** UMIN Clinical Trials Registry (UMIN-CTR): UMIN000028019

## 1. Introduction

Delayed oxygen supply to organs in critically ill patients, including those with septic shock, contributes to high mortality [1, 2]. Timely administration of vasopressors, such as noradrenaline, to patients with septic shock is crucial to facilitate organ perfusion. Noradrenaline is usually administered via central venous catheters (CVCs) [3]. However, CVC insertion can cause arterial puncture and pneumothorax, and the time required for CVC insertion can potentially delay the prompt initiation of noradrenaline [4–6]. In contrast, administering noradrenaline via a peripheral intravenous catheter (PIVC) can avoid approximately 30%–50% of CVC insertions [7–9]. Therefore, the Surviving Sepsis Campaign Guidelines 2021 (SSCG2021) allow administering vasopressors through PIVCs to avoid delays associated with CVCs [2]. Administration of noradrenaline through PIVCs can considerably expedite its delivery compared to that using CVCs [5]; however, complications, such as phlebitis and extravasation, leading to tissue injury occur in approximately 3%–12% of cases [3, 10]. Therefore, identifying the safest dosing regimen to avoid complications of PIVC-administered noradrenaline is crucial.

The potential risk factors for complications arising from PIVC-administered noradrenaline include its concentration, infusion rate, duration, and dose [10–12]. Current guidelines, such as Infusion Therapy Standards of Practice (ninth edition) published from the Infusion Nurses Society, mention the need for caution when administering vasoconstrictive drugs via PIVCs but do not provide specific recommendations on administration methods or dose limitations [13]. Similarly, the SSCG2021 recommends early noradrenaline administration via PIVCs but does not specify the duration or maximum dose that can be tolerated [2]. However, few studies have examined the relationship between these risk factors and complication development [5, 14, 15]. A systematic review exploring the duration of PIVC-administered vasopressors indicated that local tissue damage occurs in patients receiving vasopressors for greater than 6–24 h [16].

Most studies investigating the relationship between the risk factors for vasopressor drugs, such as noradrenaline (concentration, rate, duration, and dose), and PIVC complications have relied solely on the univariate analysis, which does not control for confounding factors [12, 16–18]. In addition, the influence of the drug was treated as a time-independent factor, potentially misrepresenting the true clinical nature where adrenaline dosage may vary over several hours or days. As complications associated with noradrenaline administration may be interrelated with the infusion rate and duration, examining each risk factor independently for clinical application is insufficient. Therefore, the impact of the total noradrenaline dose over the PIVC dwells on the development of phlebitis, a common complication characterized by inflammation of the vein wall that can cause pain and discomfort and potentially lead to skin necrosis, should be evaluated, while considering the influence of rate and duration. However, no study has examined the correlation between the total dose and PIVC-related complications, with the drug considered a time-dependent factor (eFigure 1). This concept is particularly interesting in critically ill patients, as their condition and the duration of drug infusion may significantly impact the occurrence and severity of PIVC-related complications. Investigating this relationship could provide valuable insights into optimizing PIVC management in this patient population.

Therefore, we aimed to investigate the relationship between the total dose of noradrenaline administered via PIVCs and the development of phlebitis using a multiple Cox regression model to allow for the influence of drug administration as a time-dependent factor.

## 2. Materials and Methods

### 2.1. Study Design and Setting

Post hoc analysis using prospectively collected clinical data from the AMOR-VENUS database, incorporating prospective multicenter cohort data from 22 centers and 23 intensive care units (ICUs) in Japan [19]. This study adhered to the Strengthening the Reporting of Observational Studies in Epidemiology guidelines [20].

### 2.2. Ethical Approval

This post hoc analysis was approved by the Jichi Medical University Saitama Medical Center (Approval number: S21-006). The requirement for informed consent was waived, and an opt-out recruitment method was employed. This study was conducted in accordance with the principles of the Declaration of Helsinki.

### 2.3. Study Participants and PIVC Inclusion

Data from the AMOR-VENUS database on all consecutive PIVCs newly inserted in ICU in patients aged ≥ 18 years were included. The selection and exclusion criteria are available in a previously published AMOR-VENUS study [19]. In addition to previous exclusions, the following PIVCs were excluded if data were missing for: (1) intravenous medication administration method (intermittent or continuous), (2) catheter removal time, and (3) medication administration time.

### 2.4. Data Collection

Data collected from the AMOR-VENUS database to demonstrate the association between noradrenaline administration and the occurrence of phlebitis included ICU characteristics (provision of standard medication administration procedures in the ICU and provision of nurse education regarding PIVC catheter management), patient characteristics (age, sex, height, weight, Acute Physiology and Chronic Health Evaluation [APACHE] II score [21], Simplified Acute Physiology Score II [22], Sequential Organ Failure Assessment score [23], Charlson comorbidity index [24], route and type of ICU admission, category of ICU admission, presence of CVC and peripherally inserted central catheter [PICC] insertions, and sepsis at ICU admission [25]), PIVC characteristics (medical staff who inserted the catheter, insertion site, catheter material and gauge, use of ultrasound, number of insertion attempts, dressing method, infection during catheterization, and catheter dwell time), information on drugs administered via PIVC during ICU stay, and phlebitis-related outcomes.

### 2.5. Exposure and Definitions

Exposure was defined as the total amount of continuously administered noradrenaline. All noradrenaline doses in this study are expressed as noradrenaline base (not as noradrenaline tartrate or bitartrate salt). The total amount of administered noradrenaline was calculated as the cumulative sum of the quantities administered per unit time (h) from one PIVC's insertion to removal (eFigure 2).

### 2.6. Outcomes

The primary endpoint was phlebitis, which was defined according to the phlebitis scale developed by the Infusion Nurses Society [26]. Phlebitis was diagnosed if all five clinical signs were observed, and it was categorized into four grades (e-Tables 1 and 2) [26]. If a patient was unable to evaluate symptoms such as pain, trained nurses at each study site assessed the pain levels using the Face Scale or Behavioral Pain Scale [27]. To reduce information bias, pilot training was conducted to enable an accurate diagnosis of phlebitis. Additionally, specialized clinical researchers trained at the central facility monitored the accuracy of phlebitis diagnosis throughout the study. The accuracy of catheter insertion site information was verified by transmitting phlebitis images from each study site to the data management center during the first month after data collection. The primary objective of the study was to examine the association between the total noradrenaline dose and the incidence of phlebitis.

### 2.7. Directed Acyclic Graphs (DAGs)

To clarify the causal relationship between noradrenaline administration and the occurrence of phlebitis, the confounding factors that exist between the two were organized using DAGs. The DAGs were constructed based on the knowledge from previously reported papers and consensus within the research team [19, 28–38]. Briefly, the creation process was as follows: (1) the potential risk factors that may contribute to the association between the exposure factor of noradrenaline administration and the outcome of phlebitis occurrence were listed, (2) the exposure factor, outcome, and listed factors were connected with one-way arrows representing their relationships, (3) factors to adjust for were selected, and the minimum factors that close the causal pathway were extracted, and (4) the extracted factors were designated as confounders involved in the causal relationship between noradrenaline administration and phlebitis. The confounding variables were selected based on the DAGs extracted using DAGitty, a browser-based environment for creating, editing, and analyzing causal diagrams [39] (Accessed February 23, 2024. https://www.dagitty.net) (eTable 3 and eFigure 3). Ultimately, the following factors were extracted as confounding factors from four levels of data hierarchy: ICU characteristics (standardized medication management procedures), patient-level variables (presence of CVC or PICC during PIVC insertion), catheter-level variables (catheter insertion site), and drug variables (the total administered nicardipine dose).

### 2.8. Statistical Analyses

Patient, catheter, and drug characteristics were described using the means and standard deviations, or medians and interquartile ranges (IQRs) for continuous variables and percentages for categorical variables. Two-sample *t*-tests or Mann–Whitney *U* tests were used to compare continuous variables, whereas chi-squared or Fisher's exact tests were applied for categorical variables, as appropriate. The association between the timing of phlebitis and the total administered noradrenaline dose was evaluated using the multilevel multiple Cox regression model, accounting for correlations within patients across catheters and institutions while considering the influence of time-dependent variables. The origin of this study in this Cox regression model was the time of PIVC insertion in the ICU. Censoring was defined as the removal of PIVC or discharge from the ICU with the PIVC in situ. The primary multiple Cox regression model included four confounding variables from four levels based on the DAG (eTable 3 and eFigure 3) as shown in the “*Directed acyclic graphs*” section (Model 1). Among the exposure factor (noradrenaline administration dose) and the four confounding factors, the total administered noradrenaline dose, total administered nicardipine dose, and the timing of CVC/PICC insertion/removal were incorporated into the analysis model as time-dependent factors. The model accounted for the hourly administration of each drug and the presence or absence of CVC/PICC insertion/removal events within each hour (eFigure 2). The results of the multilevel multiple Cox regression model using time-dependent variables are presented as hazard ratios (HRs) and 95% confidence intervals (CIs). Nonlinear relationships between the total noradrenaline dose and incidence of phlebitis were considered, and the results were visualized using spline curves, thereby assessing the association between exposure and outcomes with overall HRs and 95% CIs. The number of degrees of freedom for the smoothing spline of the total noradrenaline dose was three.

Missing values were imputed using the “mice” package with multiple imputations (*m* = 5, maxit = 100, method = “pmm,” and seed = 500) [40]. Imputed measures were completed using all predictors, outcomes, and other covariates. For sensitivity analysis, in addition to those extracted from DAG, another model that included other potential risk factors for phlebitis, such as age, sex, body mass index, APACHE II, insertion site, catheter gauge, and the total dose of amiodarone, ampicillin/sulbactam, cefepime, ceftriaxone, meropenem, potassium, peripheral parenteral nutrition, propofol, and vancomycin (Model 2) [19, 28–38], was analyzed using a multilevel multiple Cox regression model considering time-dependent variables, and smoothing spline was drawn to visualize the results. The validity of the included factors was assessed by testing the interactions and examining their multicollinearity. Multicollinearity among the factors was assessed using variance inflation factors, with values > 10 indicating multicollinearity. If multicollinearity was observed, one variable was excluded from the model. Additionally, the efficacy of the multilevel multiple Cox regression models with time-dependent variables used in this study (Models 1 and 2) was examined by comparing them with a standard Cox regression model. The same variables used in the main and sensitivity analyses were incorporated (Models 3 and 4), and the exposure factors were separately examined for both the total dose of noradrenaline and the presence or absence of noradrenaline administration (binary variables). In all statistical analyses, *p* < 0.05 was considered statistically significant. These analyses were conducted using R-4.2.1 (R Foundation for Statistical Computing, Vienna, Austria).

## 3. Results

### 3.1. Patient and PIVC Selection

**Figure 1:**
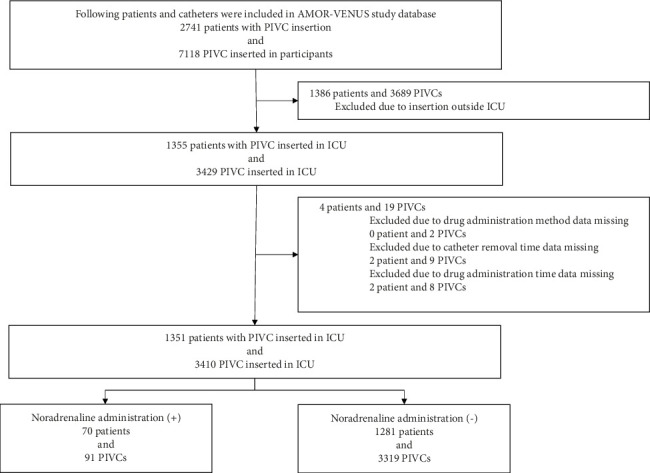
Patient flowchart. ICU, intensive care unit; PIVC, peripheral intravenous catheter.

Of the 2741 patients and 7118 PIVCs registered in the AMOR-VENUS study, 1386 patients and 3689 PIVCs were excluded (Figure 1). Additionally, to analyze the effect of the administered drugs as time-dependent variables, patients and PIVCs with missing data on drug administration methods, catheter removal times, and medication times were excluded, resulting in the inclusion of 1351 patients and 3410 PIVCs in the analysis.

### 3.2. Patient Characteristics

Noradrenaline was administered to 70 patients (5.2%) through 91 PIVCs (2.6%). The characteristics of patients who received noradrenaline during their ICU stay compared with those who did not are shown in Table 1.

### 3.3. PIVC Characteristics

Table 2 presents the characteristics of PIVCs with and without noradrenaline administration. The median and IQR of catheter insertion duration were 46.2 h (21.3–82.9) overall, 41.3 h (23.8–78.3) in the noradrenaline administration group, and 46.3 h (21.3–83.0) in the non-noradrenaline administration group.

### 3.4. Noradrenaline Administration and Phlebitis Occurrence

**Figure 2:**
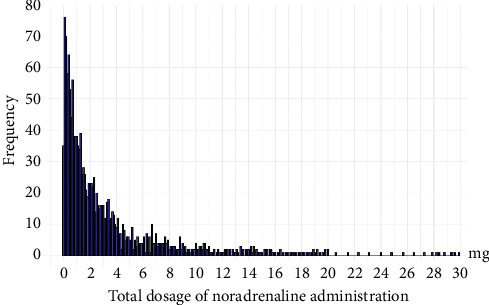
Histogram of total noradrenaline dose and frequency. The *x*-axis represents the total administered noradrenaline dose (mg), and the *y*-axis represents the frequency per unit dose.

Table 3 shows the epidemiological characteristics of the noradrenaline administration methods in 91 PIVCs, of which 19 (20.9%) developed phlebitis. These data included the average noradrenaline infusion rate, total noradrenaline dose, duration of administration, and noradrenaline concentration. The median infusion rate and IQR for noradrenaline in the phlebitis occurrence group were 0.17 (0.09–0.20) mg/h, and the median peak infusion rate and IQR were 0.30 (0.17–0.41) mg/h. No significant difference was observed in the infusion rate between the phlebitis and nonphlebitis groups (*p*=0.50). The median and IQR for the total dose of noradrenaline in 91 PIVCs were 1.55 mg (0.68–4.03), with most doses being < 10 mg (Figure 2). The median total dose of noradrenaline and IQR for the phlebitis occurrence group was 2.61 mg (1.83–4.70), significantly higher than that of the nonoccurrence group (*p* < 0.05).

### 3.5. Time-Dependent Multilevel Multiple Cox Regression Models

**Figure 3:**
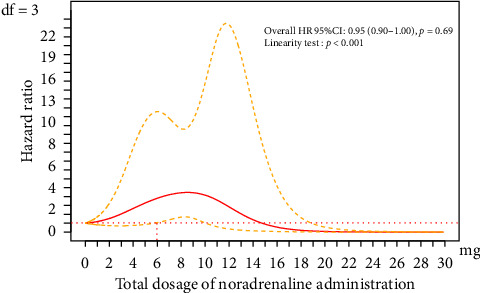
Restricted cubic spline curve with three degrees of freedom using a time-dependent multiple Cox regression model. The *x*-axis represents the total administered noradrenaline dose (mg), and the *y*-axis represents the HR for each total administered noradrenaline dose, with the reference being the total noradrenaline dose set to zero. The solid red line indicates the HR estimated using a multilevel multiple Cox regression model, treating the total dose of noradrenaline as a time-dependent factor, and the orange dashed line indicates the 95% CI (degrees of freedom: 3). The overall HR and 95% CI were 0.95 (0.90–1.00), and the linearity test result was *p* < 0.001. The covariates included in Model 1 were the presence of standardized medication management procedures, the presence of a CVC or PICC during PIVC insertion, the catheter insertion site, and the total administered nicardipine dose. CI, confidence interval; CVC, central venous catheter; HR, hazard ratio; PICC, peripherally inserted central catheter; PIVC, peripheral intravenous catheter.

No significant association was observed between the total administered dose and the occurrence of phlebitis (HR 1.06, 95% CI 0.93–1.20, *p*=0.38) (Table 4) when incorporating the impact of the drug as a time-dependent factor into the multilevel multiple Cox regression model (Model 1). However, spline curves drawn using the same analysis model revealed a significant relationship between the total noradrenaline dose and phlebitis when the total dose exceeded 6 mg within the range where most doses were distributed (< 10 mg), as the lower limit of the 95% CI exceeded the significant threshold of 1.0 (Figure 3).

### 3.6. Sensitivity Analysis and Model Validation

Sensitivity analysis was performed using Model 2, which included a different set of factors, to assess the validity of the results from Model 1; this model showed similar results (HR, 1.06; 95% CI 0.94–1.21, *p*=0.35) (Table 4, and eFigure 4). Additionally, the validity of the analytical model was verified by interaction tests and multicollinearity assessments of the factors included in Models 1 and 2 (eTables 4 and 5).

### 3.7. Standard Cox Regression Models

The results of standard Cox regression models (Models 3 and 4), which included the same factors as the covariates in Models 1 and 2 (time-dependent multilevel multiple Cox regression models), are presented in eTable 6, eFigures 5, and 6. In both models, the standard Cox regression model recognized a significant association between the total administered dose and the occurrence of phlebitis (Model 3: HR 1.10, 95% CI 1.04–1.17, Model 4: HR 1.11, 95% CI 1.05–1.18).

## 4. Discussion

The current study, using time-dependent analysis, spline curves, and DAGs for multilevel multiple Cox regression model to examine the causal inference, assessed PIVC-related phlebitis risk using the total administered drug dose. Our results suggested an increased risk of phlebitis onset when the total continuously administered noradrenaline dose exceeded 6 mg.

**Figure 4:**
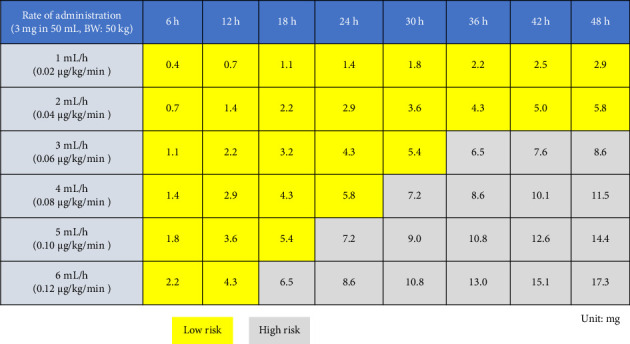
Risk determination table for the occurrence of phlebitis according to the rate and duration of noradrenaline administration. Each cell in this table represents the total administered dose calculated from the matrix of noradrenaline administration rate and duration, assuming a concentration of 60 μg/mL and a body weight of 50 kg. Yellow cells indicate cases where the total dose of noradrenaline is ≤ 6 mg, and Gray cells represent combinations where the amount exceeds 6 mg. For example, an administration rate of 5 mL/h, i.e., 0.1 μg/kg/min, suggests the possibility of administering noradrenaline without phlebitis occurrence for up to 18 h. Similarly, an administration rate of 2 mL/h, i.e., 0.04 μg/kg/min, suggests the possibility of administering noradrenaline without phlebitis occurrence for up to 48 h.

Evaluating the impact of drugs on the occurrence of phlebitis using the total administered dose may provide a more clinically relevant indicator than previous methods [10–12, 16–18]. Drug effects on complications are influenced by infusion rate, duration, and dose [10–12]; however, most studies only consider the presence or absence of drug administration [12, 16, 18], resulting in applying these findings to clinical practice has been challenging. To address this issue, the current study examined the total administered dose of the drug, which incorporates both infusion rate and duration. Evaluating the impact of drugs on phlebitis using the total administered dose as a parameter allows for risk assessment with a single parameter, regardless of changes in drug administration rate or duration. This approach enables a more clinically applicable risk assessment of exposure factors, facilitating interventions in clinical settings. For example, a matrix of infusion rates and durations within this limit can be developed for a given concentration (Figure 4), thereby elucidating which patterns affect phlebitis risk.

The identification of a total dose threshold for increased phlebitis risk, as demonstrated by the analytical approaches used in this study, enables the safe administration of noradrenaline via PIVCs if the total dose remains below the specified cutoff value. This finding has the potential to facilitate risk avoidance in clinical settings. The results of this study may suggest that limiting the total noradrenaline dose to a specific threshold using time-dependent analysis and spline curves may enable safer administration through PIVCs. This information can complement the recommendations provided in these guidelines and contribute to improving the quality of PIVC management and safety in noradrenaline administration via PIVCs in clinical practice. Integrating total dose considerations into current PIVC noradrenaline protocols could refine these guidelines further.

Moreover, the identification of a cutoff value for the relatively safe total dose of noradrenaline that can be administered via PIVCs may allow for administration up to that dose without the need for CVC insertion, potentially delaying or eliminating the need for CVC placement. Some institutions have policies that require noradrenaline to be administered primarily through CVCs [41]. In such institutions, even small amounts of noradrenaline cannot be administered until a CVC is inserted, which may impact patient outcomes, such as prolonged shock due to delayed noradrenaline administration. Conversely, in regions where noradrenaline administration via PIVCs is permitted, the results of this study may suggest that a substantial proportion of CVC insertions can be avoided by administering noradrenaline through PIVCs [7–9]. However, without a specified cutoff value for safe noradrenaline administration, the appropriateness of PIVC use for this purpose may be overestimated or underestimated. The establishment of such a safety threshold cutoff value may enable the avoidance of many CVC insertions for the purpose of noradrenaline administration. This, in turn, could lead to the prevention of serious complications associated with CVCs and contribute to improved patient outcomes.

To effectively utilize the findings of this study in clinical practice, it is crucial to accurately monitor and manage the total dose of noradrenaline administered. This necessitates the implementation of a warning system, primarily led by pharmacists. Leveraging healthcare information systems to automatically calculate and record noradrenaline dosages can also be beneficial [42]. Incorporating features such as alerts when the total dose approaches the identified cutoff value can support decision-making in clinical settings. Furthermore, when doses exceeding the threshold are required, it is recommended to replace the PIVC, switch to administration via CVC, and closely monitor for signs of phlebitis. By implementing these measures, the safety of noradrenaline administration through PIVCs can be enhanced, potentially leading to improved outcomes for patients in shock.

## 5. Limitations

The sample size of PIVCs used for noradrenaline was relatively small, and the cutoff value for the total noradrenaline dose that significantly risks phlebitis may change with a larger sample size. Second, the total administered dose, calculated from infusion rate and duration, may be an oversimplified parameter to estimate the association with phlebitis, as different rates and durations of the same total dose could have varying risks. However, treating exposure as a time-dependent factor in the analysis model partially addressed this issue. The appropriateness of the total dose remains unclear without studies accurately evaluating the impact of drug administration; however, our results suggest one possibility. Finally, the analysis model in this study may have had insufficient confounding factors, which were limited to using DAGs. While DAGs have gained attention recently [43–46], in the emergency and intensive care fields, various factors affect patient outcomes, increasing DAG complexity, and our DAGs may not have adequately evaluated these relationships. Nevertheless, a sensitivity analysis including additional phlebitis risk factors yielded similar results, suggesting robustness.

## 6. Conclusions

This study suggests that PIVC phlebitis risk increases when the total dose of continuous noradrenaline administered exceeds 6 mg. Further investigation is needed to determine the appropriateness of using less than this total dose to prevent phlebitis in PIVCs.

## Figures and Tables

**Table 1:** Patient characteristics at ICU admission with or without noradrenaline administration.

Variables	Noradrenaline (+) *N* = 70 (5.2%)	Noradrenaline (−) *N* = 1281 (94.8%)	*p* value
Age, mean (SD), years	69.4 (12.3)	68.5 (15.2)	0.53
Gender, male (*n*, %)	47 (67.1%)	803 (62.7%)	0.53
Body height, mean (SD), cm^a^	160 (8.1)	161 (9.9)	0.72
Body weight, mean (SD), kg^b^	58.5 (12.7)	59.7 (14.9)	0.45
BMI, mean (SD)^b^	22.7 (4.4)	22.9 (4.5)	0.67
APACHE II, mean (SD)^c^	22.3 (9.0)	17.7 (7.9)	< 0.001
SAPS II, mean (SD)^c^	51.7 (20.7)	40.4 (19.3)	< 0.001
SOFA, mean (SD)^c^	8.3 (3.3)	6.0 (3.7)	< 0.001
Charlson comorbidity index, mean (SD)	4.4 (2.6)	4.4 (2.6)	0.91
ICU admission from (*n*, %)			< 0.001
Operation room	11 (15.7%)	594 (46.4%)	
Emergency room	39 (55.7%)	472 (36.8%)	
General ward	14 (20.0%)	166 (13.0%)	
Outpatients	1 (1.4%)	5 (0.4%)	
Transfer from other hospital	5 (7.1%)	44 (3.4%)	
Type of admission to ICU (*n*, %)			< 0.001
Elective surgical	5 (7.1%)	377 (29.4%)	
Emergency surgical	6 (8.6%)	217 (16.9%)	
Medical	59 (84.3%)	687 (53.6%)	
ICU admission category (*n*, %)^d^			0.03
Cardiology	14 (20.3%)	479 (37.6%)	
Pulmonary	18 (26.1%)	165 (13.0%)	
Gastrointestinal	9 (13.0%)	174 (13.7%)	
Neurology	10 (14.5%)	185 (14.5%)	
Trauma	4 (5.8%)	49 (3.9%)	
Urology	0 (0%)	17 (1.3%)	
Gynecology	0 (0%)	14 (1.1%)	
Skin/tissue	1 (1.5%)	22 (1.7%)	
Others	13 (18.8%)	168 (13.2%)	
Sepsis at ICU admission (*n*, %)			< 0.001
Sepsis	13 (18.6%)	77 (6.0%)	
Septic shock	19 (27.1%)	115 (9.0%)	
CVC/PICC insertion^∗^	30 (42.9%)	494 (38.6%)	0.55
Phlebitis (*n*, %)	12 (17.1%)	90 (7.0%)	0.004

*Note:* Missing data: ^a^*n* = 1 (noradrenaline (+) = 1, noradrenaline (−) = 0), ^b^*n* = 2 (noradrenaline (+) = 1, noradrenaline (−) = 1), ^c^*n* = 59 (noradrenaline (+) = 0, noradrenaline (−) = 59), ^d^*n* = 9 (noradrenaline (+) = 1, noradrenaline (−) = 8).

Abbreviations: APACHE, acute physiology and chronic health evaluation; BMI, body mass index; CVC, central venous catheter; ICU, intensive care unit; PICC, peripheral-inserted central venous catheter; PIVC, peripheral intravenous catheter; SAPS, simplified acute physiology score; SD, standard deviation; SOFA, sequential organ failure assessment.

^∗^Proportion of CVC or PICC insertion during ICU admission.

**Table 2:** All peripheral intravenous catheter characteristics during insertion with or without noradrenaline administration.

Variables	Noradrenaline (+) *N* = 91 (2.7%)	Noradrenaline (−) *N* = 3319 (97.3%)	*p* value
Catheter inserted by (*n*, %)^a^			0.61
Doctor	10/76 (13.2%)	276/2592 (10.6%)	
Nurse	66/76 (86.8%)	2316/2592 (89.4%)	
Inserted site (*n*, %)			0.85
Upper arm	10/91 (11.0%)	346/3292 (10.5%)	
Forearm	44/91 (48.4%)	1795/3292 (54.5%)	
Elbow	6/91 (6.6%)	157/3292 (4.8%)	
Wrist	6/91 (6.6%)	155/3292 (4.8%)	
Hand	13/91 (14.3%)	491/3292 (14.9%)	
Lower leg	8/91 (8.8%)	215/3292 (6.5%)	
Dorsal foot	4/91 (4.4%)	133/3292 (4.0%)	
Catheter material			0.58
PEU-vialon^∗^	28/91 (30.8%)	1049/3319 (31.6%)	
Polyurethane	21/91 (23.1%)	954/3319 (28.7%)	
Tetrafluoroethylene	40/91 (44.0%)	1250/3319 (37.7%)	
Others	2/91 (2.2%)	66/3319 (2.0%)	
Catheter gauge (*n*, %)^b^			0.13
≦ 18G	1/90 (1.1%)	162/3262 (5.0%)	
20G	20/90 (22.2%)	860/3262 (26.4%)	
22–24G	69/90 (76.7%)	2240/3262 (68.7%)	
Use of ultrasonography (*n*, %)^c^	1/74 (1.4%)	57/2552 (2.2%)	0.91
Number of attempts at insertion (*n*, %)^d^			0.97
1	60/74 (81.1%)	2051/2535 (80.9%)	
2	8/74 (10.8%)	304/2535 (12.0%)	
3	4/74 (5.4%)	126/2535 (5.0%)	
≧ 4	2/74 (2.7%)	54/2535 (2.1%)	
Dressing (*n*, %)^e^			0.89
Sterile	90/91 (98.9%)	3221/3289 (97.9%)	
Nonsterile	1/91 (1.1%)	68/3289 (2.1%)	
Any infection during catheter dwell (*n*, %)	45/91 (49.5%)	756/3319 (22.8%)	< 0.001
CVC/PICC insertion^∗∗^	29 (31.9%)	1111 (33.5%)	0.82
Duration of catheter dwell, median (IQR), (h)	41.3 (23.8–78.3)	46.3 (21.3–83.0)	0.65
Phlebitis (*n*, %)	19/91 (20.9%)	293/3319 (8.8%)	< 0.001

Abbreviations: CVC, central venous catheter; IQR, interquartile range, PICC, peripheral-inserted central venous catheter.

^∗^PEU-vialon: polyetherurethane without leachable additives.

^∗∗^Proportion of CVC or PICC insertion during ICU admission.

^a^Missing data: *n* = 742.

^b^Missing data: *n* = 58.

^c^Missing data: *n* = 784.

^d^Missing data: *n* = 801.

^e^Missing data: *n* = 3.

**Table 3:** Details of information on noradrenaline administrated via PIVC with or without phlebitis.

	Total *N* = 91	Phlebitis (+) *N* = 19 (20.9%)	Phlebitis (−) *N* = 72 (79.1%)	*p* value
Rate of noradrenaline administration				
Median value of each catheter				
Median (IQR) (mg/h)	0.18 (0.10–0.30)	0.17 (0.09–0.20)	0.18 (0.12–0.30)	0.50
Maximum (mg/h)	1.2	0.36	1.2	—
Maximum value of each catheter				
Median (IQR) (mg/h)	0.30 (0.15–0.47)	0.30 (0.17–0.41)	0.27 (0.14–0.48)	0.94
Maximum (mg/h)	8	8	2	—
Total dosage of noradrenaline administration				
Median (IQR) (mg)	1.55 (0.68–4.03)	2.61 (1.83–4.70)	1.33 (0.51–3.69)	0.043
Maximum (mg)	29.9	20.0	29.9	—
Duration of noradrenaline administration				
Median (IQR) (h)	8.8 (2.9–20.5)	20.5 (8.5–29.6)	7.5 (2.2–16.8)	0.005
Maximum (h)	100.0	100.0	57.8	—
Concentration of noradrenaline administration				
Median (IQR) (mg/mL)	0.05 (0.03–0.06)	0.03 (0.03–0.06)	0.06 (0.03–0.06)	0.18
Maximum (mg/mL)	0.12	0.07	0.12	—

Abbreviations: IQR, interquartile range; PIVC, peripheral intravenous catheter.

**Table 4:** Multilevel multiple Cox regression models with time-dependent covariates (Model 1 and Model 2).

	HR	95% CI	*p* value
Model 1^∗^			
Total dosage of noradrenaline administration (mg)	1.06	0.93–1.20	0.38
Model 2^∗∗^			
Total dosage of noradrenaline administration (mg)	1.06	0.94–1.21	0.35

Abbreviations: APACHE, Acute Physiology and Chronic Health Evaluation; CI, confidence interval; CVC, central venous catheter; HR, hazard ratio; PICC, peripherally inserted central catheter; PPN, peripheral parenteral nutrition.

^∗^Covariates included in Model 1: The presence of standardized medication management procedures, presence of a CVC or PICC during PIVC insertion, site of catheter insertion, and total administered dose of nicardipine.

^∗∗^Covariates included in Model 4: Age, sex, body mass index, APACHE II score, insertion site, catheter gauge, CVC/PICC insertion, drug administration standardization, and total dosage of the following drugs: amiodarone, ampicillin/sulbactam, cefepime, ceftriaxone, meropenem, nicardipine, potassium, PPN, propofol, and vancomycin.

## Data Availability

Research data are not shared.

## Supporting Information

eMethod: Phlebitis definition and measurement.

eTable 1: The definition of phlebitis (Infusion Nurse Society).

eTable 2: The definition of each element of the Infusion Nurse Society phlebitis definition.

eTable 3: Model code of causal interaction between noradrenaline administration and occurrence of phlebitis.

eTable 4: Interaction between noradrenaline administration and the other variables on the occurrence of phlebitis.

eTable 5: Variance Inflation Factor for the covariates included in Models 1 and 2.

eTable 6: Multilevel standard Cox regression models with time-independent covariates (Model 3 and Model 4).

eFigure 1: Time-dependent and non–time-dependent factors on the occurrence of phlebitis.

eFigure 2: Example data structure for Cox regression models considering time-dependent confounding factors.

eFigure 3: Directed acyclic graph related to the causal relationship between noradrenaline administration and phlebitis development.

eFigure 4: Restricted cubic spline curve with 3 degrees of freedom using a time-dependent multiple Cox regression model (Model 2).

eFigure 5: Restricted cubic spline curve with 3 degrees of freedom using the standard Cox regression model (Model 3).

eFigure 6: Restricted cubic spline curve with 3 degrees of freedom using the standard Cox regression model (Model 4).
