# Supplementary material for: Impact of Noradrenaline Administration Dosage on the Occurrence of Peripheral Intravenous Catheter–Related Venous Phlebitis in Critically Ill Patients Using a Time-Dependent Multilevel Cox Regression Model
Source: Emerg Med Int. 2025 May 6;2025:4457109. doi: 10.1155/emmi/4457109 (PMC12074845; doi:10.1155/emmi/4457109)
Supplement: Supporting Information — Additional supporting information can be found online in the Supporting Information section. [file 4457109.f1.docx]

**Supplemental digital content**

**Title**

Impact of Noradrenaline Administration Dosage on the Occurrence of Peripheral Intravenous Catheter-Related Venous Phlebitis in Critically Ill Patients Using a Time-Dependent Multilevel Cox Regression Model

**Contents**

| **eMethods** | |
| --- | --- |
| **eMethod** | **Phlebitis Definition and Measurement** |
| **eTables** | |
| **eTable 1** | **The definition of phlebitis (Infusion Nurse Society)** |
| **eTable 2** | **The definition of each element of Infusion Nurse Society phlebitis definition** |
| **eTable 3** | **Model code of causal interaction between noradrenaline administration and occurrence of phlebitis** |
| **eTable 4** | **Interaction between noradrenaline administration and the other variables on the occurrence of phlebitis** |
| **eTable 5** | **Variance Inflation Factor for the covariates included in Models 1 and 2** |
| **eTable 6** | **Multilevel standard Cox regression models with time-independent covariates (Model 3 and Model 4)** |
| **eFigures** | |
| **eFigure 1** | **Time-dependent and non-time-dependent factors on the occurrence of phlebitis** |
| **eFigure 2** | **Example data structure for Cox regression models considering time-dependent confounding factors** |
| **eFigure 3** | **Directed acyclic graph related to the causal relationship between noradrenaline administration and phlebitis development** |
| **eFigure 4** | **Restricted cubic spline curve with 3 degrees of freedom using time-dependent multiple Cox regression model (Model 2)** |
| **eFigure 5** | **Restricted cubic spline curve with 3 degrees of freedom using standard Cox regression model (Model 3)** |
| **eFigure 6** | **Restricted cubic spline curve with 3 degrees of freedom using standard Cox regression model (Model 4)** |

**eMethod**

*Phlebitis Definition and Measurement*

Phlebitis was diagnosed if all five clinical signs were observed, and it was categorized into four grades (see e-Tables 1 and 2 in the Additional File) [1]. If a patient was unable to evaluate symptoms such as pain, trained nurses at each study site assessed the pain levels using the Face Scale or Behavioral Pain Scale [2]. To reduce information bias, pilot training was conducted to enable an accurate diagnosis of phlebitis. Additionally, specialized clinical researchers trained at the central facility monitored the accuracy of phlebitis diagnosis throughout the study. The accuracy of catheter insertion site information was verified by transmitting phlebitis images from each study site to the data management center during the first month after data collection.

References

1. Infusion Nurses Society. Infusion nursing standards of practice. J Infus Nurs. 2006 Jan-Feb; 29(1 Suppl):S1–S92.
2. Payen JF, Bru O, Bosson JL, et al.:Assessing pain in critically ill sedated patients by using a behavioral pain scale. Crit Care Med 29:2258-2263, 2001

**eTable 1 The definition of phlebitis (Infusion Nurse Society)**

| Grade | Criteria |
| --- | --- |
| 0 | No clinical symptoms |
| 1 | Erythema at access site with or without pain |
| 2 | Pain at access site with erythema and/or edema |
| 3 | Pain at access site with erythema and/or edema, streak formation, palpable venous cord |
| 4 | Pain at access site with erythema and/or edema, or palpable venous cord > 1 inch, purulent drainage |

**eTable 2 The definition of each element of Infusion Nurse Society phlebitis definition**

| Element | definition |
| --- | --- |
| Pain | Pain around PIVC insertion site |
| Erythema | Redness around PIVC insertion site |
| Edema | Swelling around PIVC insertion site |
| Streak formation | Redness along blood vessels at PIVC insertion site |
| Palpable venous cord | Induration along blood vessels at PIVC insertion site |

PIVC, peripheral intravenous catheter

**eTable 3 Model code of causal interaction between noradrenaline administration and occurrence of phlebitis**

dag {

bb="-11.74,-10.045,10.825,8.672"

"CI/ICH" [pos="9.524,6.619"]

"CVC/PICC" [adjusted,pos="3.965,0.824"]

"Cardiogenic " [pos="9.405,2.936"]

"Catheter material" [pos="-10.032,-1.181"]

"Catheter mobility" [latent,pos="-7.717,-0.616"]

"Catheter site" [adjusted,pos="-3.548,0.581"]

"Catheter size" [pos="-6.781,0.993"]

"Difficult of visibility of vein" [pos="-2.513,4.747"]

"Difficult to palpate blood vessels" [pos="-1.803,7.223"]

"Disease severity" [pos="6.951,2.272"]

"Disinfect of insertion site" [pos="-8.682,2.207"]

"Dressing material" [pos="-9.894,0.372"]

"Drug standard" [adjusted,pos="-4.406,-9.381"]

"Gastrointestinal bleeding" [pos="5.709,5.744"]

"Hand hygiene" [pos="-7.540,5.532"]

"ICU pharmacist" [pos="-4.376,-7.751"]

"Indwelling time" [pos="-4.521,-0.955"]

"Intra-abdominal disease" [pos="7.897,7.374"]

"Location of insertion" [pos="-1.271,1.306"]

"Ns education" [pos="-7.540,-7.630"]

"Number of insertion" [pos="-7.481,7.253"]

"Physical movement" [pos="-5.027,2.363"]

"Stimulation of blood vessels" [latent,pos="-2.306,3.057"]

"Vessel Mobility" [pos="-4.051,6.468"]

AVP [pos="6.093,-6.905"]

Adrenaline [pos="1.420,-6.845"]

Age [pos="3.047,2.151"]

Albumin [pos="7.986,-6.966"]

Amiodarone [pos="3.964,-6.875"]

Antibiotics [pos="1.450,-8.837"]

Antisepsis [pos="-10.498,3.751"]

BMI [pos="4.733,4.114"]

DEX [pos="6.034,-4.853"]

DOA [pos="3.846,-8.868"]

DOB [pos="10.145,-4.883"]

Institution [pos="-10.143,-9.381"]

MV [pos="4.703,2.272"]

Midazoram [pos="1.509,-4.762"]

Nicardipine [adjusted,pos="8.075,-4.883"]

Noradrenaline [exposure,pos="-1.921,-2.619"]

Opioid [pos="4.023,-4.792"]

PPI [pos="7.956,-8.777"]

PPN [pos="10.027,-8.747"]

Person [pos="-10.498,7.223"]

Phlebitis [outcome,pos="1.687,-2.679"]

Propofol [pos="9.967,-6.936"]

Sepsis [pos="9.494,4.868"]

Sex [pos="3.077,4.114"]

Shock [latent,pos="6.418,4.053"]

Steroid [pos="6.093,-8.837"]

Trauma [pos="7.986,5.049"]

US [pos="-10.439,5.472"]

hypovolemia [pos="3.875,7.374"]

"CI/ICH" -> "Disease severity"

"CI/ICH" -> Nicardipine

"CVC/PICC" -> AVP

"CVC/PICC" -> Adrenaline

"CVC/PICC" -> DOA

"CVC/PICC" -> DOB

"CVC/PICC" -> Noradrenaline

"CVC/PICC" -> PPN

"Cardiogenic " -> DOA

"Cardiogenic " -> DOB

"Cardiogenic " -> Shock

"Catheter material" -> "Catheter mobility"

"Catheter material" -> "Stimulation of blood vessels"

"Catheter mobility" -> "Stimulation of blood vessels"

"Catheter site" -> "Catheter size"

"Catheter site" -> AVP

"Catheter site" -> Adrenaline

"Catheter site" -> DOA

"Catheter site" -> DOB

"Catheter site" -> Nicardipine

"Catheter site" -> Noradrenaline

"Catheter site" -> Phlebitis

"Catheter size" -> "Stimulation of blood vessels"

"Difficult of visibility of vein" -> "Catheter site"

"Difficult of visibility of vein" -> "Number of insertion"

"Difficult of visibility of vein" -> Person

"Difficult of visibility of vein" -> US

"Difficult to palpate blood vessels" -> "Catheter site"

"Difficult to palpate blood vessels" -> "Number of insertion"

"Difficult to palpate blood vessels" -> Person

"Difficult to palpate blood vessels" -> US

"Disease severity" -> "Catheter size"

"Disease severity" -> Amiodarone

"Disease severity" -> PPI

"Disinfect of insertion site" -> Phlebitis

"Dressing material" -> "Catheter mobility"

"Dressing material" -> "Disinfect of insertion site"

"Drug standard" -> Noradrenaline

"Gastrointestinal bleeding" -> PPI

"Gastrointestinal bleeding" -> PPN

"Gastrointestinal bleeding" -> hypovolemia

"Hand hygiene" -> "Disinfect of insertion site"

"Hand hygiene" <-> "Ns education"

"ICU pharmacist" -> "Drug standard"

"Indwelling time" -> "Catheter mobility"

"Indwelling time" -> "Disinfect of insertion site"

"Intra-abdominal disease" -> "Disease severity"

"Intra-abdominal disease" -> PPN

"Intra-abdominal disease" -> Sepsis

"Location of insertion" -> "Catheter site"

"Location of insertion" -> "Catheter size"

"Location of insertion" -> "Disinfect of insertion site"

"Location of insertion" -> "Number of insertion"

"Location of insertion" -> Person

"Ns education" -> "Disinfect of insertion site"

"Number of insertion" -> "Stimulation of blood vessels"

"Physical movement" -> "Catheter mobility"

"Physical movement" -> "Disinfect of insertion site"

"Stimulation of blood vessels" -> Phlebitis

"Vessel Mobility" -> "Number of insertion"

AVP -> Phlebitis

Adrenaline -> Phlebitis

Age -> "Difficult of visibility of vein"

Age -> "Difficult to palpate blood vessels"

Age -> BMI

Albumin -> Phlebitis

Amiodarone -> Phlebitis

Antibiotics -> Person

Antibiotics -> Phlebitis

Antisepsis -> "Disinfect of insertion site"

BMI -> "Catheter site"

BMI -> "Catheter size"

BMI -> "Difficult of visibility of vein"

BMI -> "Difficult to palpate blood vessels"

BMI -> "Vessel Mobility"

DEX -> Phlebitis

DOA -> Phlebitis

DOB -> Phlebitis

Institution -> "Catheter material"

Institution -> "Dressing material"

Institution -> "Drug standard"

Institution -> "ICU pharmacist"

Institution -> "Ns education"

Institution -> Antisepsis

MV -> "Disease severity"

MV -> DEX

MV -> Midazoram

MV -> Opioid

MV -> Propofol

Midazoram -> Phlebitis

Nicardipine -> AVP

Nicardipine -> Adrenaline

Nicardipine -> DOA

Nicardipine -> DOB

Nicardipine -> Noradrenaline

Nicardipine -> Phlebitis

Noradrenaline -> AVP

Noradrenaline -> Adrenaline

Noradrenaline -> DOA

Noradrenaline -> DOB

Noradrenaline -> PPN

Noradrenaline -> Phlebitis

Noradrenaline -> Steroid

Opioid -> Phlebitis

PPI -> Phlebitis

PPN -> Phlebitis

Person -> "Number of insertion"

Person -> US

Propofol -> Phlebitis

Sepsis -> "Disease severity"

Sepsis -> Antibiotics

Sepsis -> Shock

Sepsis -> hypovolemia

Sex -> Phlebitis

Shock -> "CVC/PICC"

Shock -> "Disease severity"

Shock -> Albumin

Shock -> DOA

Shock -> Nicardipine

Steroid -> Phlebitis

Trauma -> Opioid

Trauma -> hypovolemia

US -> "Number of insertion"

hypovolemia -> "Catheter size"

hypovolemia -> Albumin

hypovolemia -> Shock

}

**eTable 4 Interaction between noradrenaline administration and the other variables on the occurrence of phlebitis**

| Variables | p value |
| --- | --- |
| Age | 0.48 |
| Gender | 0.47 |
| Body mass index | 0.93 |
| APACHE II | 0.98 |
| Inserted Site | 0.13 |
| Catheter gauge | 0.29 |
| CVC/PICC insertion | 0.26 |
| Drug administration standardization | 0.12 |
| Amiodarone | NA |
| Ampicillin/sulbactam | 0.99 |
| Cefepime | 0.99 |
| Ceftriaxone | 0.85 |
| Meropenem | 0.65 |
| Nicardipine | 0.99 |
| Potassium | 0.37 |
| PPN | 0.01 |
| Propofol | 0.89 |
| Vancomycin | 0.99 |

APACHE, acute physiology and chronic health evaluation; CVC, central venous catheter; PICC, peripherally inserted central catheter; PPN, peripheral parenteral nutrition

**eTable 5 Variance Inflation Factors for the covariates included in Models 1 and 2**

| Variables | Model 1 | Model 2 | Variance Inflation Factors |
| --- | --- | --- | --- |
| Age |  | ● | 1.07 |
| Sex |  | ● | 1.03 |
| Body mass index |  | ● | 1.04 |
| APACHE II |  | ● | 1.04 |
| Insertion Site | ● | ● | 1.01 |
| Catheter gauge |  | ● | 1.04 |
| CVC/PICC insertion | ● | ● | 1.04 |
| Drug administration standardization | ● | ● | 1.04 |
| Amiodarone |  | ● | 1.01 |
| Ampicillin/sulbactam |  | ● | 1.01 |
| Cefepime |  | ● | 1.01 |
| Ceftriaxone |  | ● | 1.01 |
| Meropenem |  | ● | 1.05 |
| Nicardipine | ● | ● | 1.01 |
| Potassium |  | ● | 1.00 |
| PPN |  | ● | 1.01 |
| Propofol |  | ● | 1.01 |
| Vancomycin |  | ● | 1.05 |

APACHE, acute physiology and chronic health evaluation; CVC, central venous catheter; PICC, peripherally inserted central catheter; PPN, peripheral parenteral nutrition

**eTable 6 Multilevel standard Cox regression models with time-independent covariates (Model 3 and Model 4)**

|  | HR | 95% CI | p value |
| --- | --- | --- | --- |
| Model 3* |  |  |  |
| Total dosage of noradrenaline administration, mg | 1.10 | 1.04-1.17 | 0.002 |
| Noradrenaline administration (as binary variable) | 2.51 | 1.51-4.17 | <0.001 |
| Model 4** |  |  |  |
| Total dosage of noradrenaline administration, mg | 1.11 | 1.05-1.18 | <0.001 |
| Noradrenaline administration (as binary variable) | 2.62 | 1.57-4.38 | <0.001 |

APACHE, Acute Physiology and Chronic Health Evaluation; CI, confidence interval; CVC, central venous catheter; HR, hazard ratio; PICC, peripherally inserted central catheter; PPN, peripheral parenteral nutrition

*Covariates included in Model 3: the presence of standardized medication management procedures, presence of a CVC or PICC during PIVC insertion, site of catheter insertion, and total administered dose of nicardipine

** Covariates included in Model 4: age, sex, body mass index, APACHE II score, insertion site, catheter gauge, CVC/PICC insertion, drug administration standardization, and total dosage of the following drugs: amiodarone, ampicillin/sulbactam, cefepime, ceftriaxone, meropenem, nicardipine, potassium, PPN, propofol, and vancomycin

**eFigure 1 Time-dependent and non-time-dependent factors affecting the occurrence of phlebitis**

The exposure time points of factors affecting the occurrence of PIVC-related phlebitis, from PIVC insertion to removal, are indicated. ICU, patient, and PIVC-related factors were exposed before PIVC insertion and are time-independent factors whose exposure levels do not change until PIVC removal. In contrast, drugs and CVC/PICC insertion, which are exposed between PIVC insertion and removal with varying start and end times for each factor, are time-dependent factors whose exposure duration affects the outcome differently.

CVC, central venous catheter; ICU, intensive care unit; PICC, peripherally inserted central catheter; PIVC, peripheral intravenous catheter.

**eFigure 2 Example data structure of the Cox regression model considering time-dependent confounding factors**

This Figure shows part of the data structure used in the analysis. For each PIVC (column B), the time from catheter insertion to removal was divided into 1-h intervals (column C: drug administration start time, column D: drug administration end time); the presence or absence of exposure to each factor within that unit time was stored in column F. The presence or absence of outcome occurrence within that unit time was stored in column E. The time-dependent factor of noradrenaline administration (column G: administration rate, column H: administered dose) showed changes within a unit of time, whereas the time-independent factor of age showed the same value for all time units. A multilevel multiple Cox regression model, considering the impact of time-dependent factors, was conducted using this data structure.
PIVC, peripheral intravenous catheter.

**eFigure 3 Directed acyclic graph of the causal relationship between noradrenaline administration and phlebitis development**

White circles represent confounders to be adjusted for, blue circles are ancestors of the outcome, red circles are ancestors of the exposure and outcome, and gray circles represent factors that were not measured in this study.
AVP, arginine vasopressin; BMI, body mass index; CI, cerebral infarction; CVC, central venous catheter; DEX, dexmedetomidine; DOA, dopamine; DOB, dobutamine; ICH, intracranial hemorrhage; ICU, intensive care unit; MV, mechanical ventilation; PICC, peripherally inserted central catheter; PPI, proton-pump inhibitor; PPN, peripheral parenteral nutrition; US, ultrasonography

**eFigure 4 Restricted cubic spline curve with three degrees of freedom using a time-dependent multiple Cox regression model (Model 2)**

The x-axis represents the total administered noradrenaline dose (mg), and the y-axis represents the HR for each total administered noradrenaline dose, with the reference being the total noradrenaline dose set to zero. The solid red line indicates the HR estimated using multilevel multiple Cox regression model, treating the total dose of noradrenaline as a time-dependent factor, and the orange dashed line indicates the 95% CI (degrees of freedom: 3). The overall HR and 95% CI were 0.93 (0.88–0.99), and the linearity test result was p < 0.001. The covariates included in Model 2 were age, sex, body mass index, APACHE II score, insertion site, catheter gauge, CVC/PICC insertion, drug administration standardization, and total dose of amiodarone, ampicillin/sulbactam, cefepime, ceftriaxone, meropenem, nicardipine, potassium, PPN, propofol, and vancomycin.
APACHE, Acute Physiology and Chronic Health Evaluation; CI, confidence interval; CVC, central venous catheter; HR, hazard ratio; PICC, peripherally inserted central catheter; PIVC, peripheral intravenous catheter; PPN, peripheral parenteral nutrition

**eFigure 5 Restricted cubic spline curve with three degrees of freedom using the standard Cox regression model (Model 3)**

The x-axis represents the total administered noradrenaline dose (mg), and the y-axis represents the HR for each total administered noradrenaline dose, with the reference being the total noradrenaline dose set to zero. The solid red line indicates the HR estimated using multilevel standard Cox regression model, treating the total dose of noradrenaline as a time-independent factor, and the orange dashed line indicates the 95% CI (degrees of freedom: 3). The overall HR and 95% CI were 1.11 (1.03–1.19), and the linearity test result was p < 0.01. The covariates included in Model 1 were the presence of standardized medication management procedures, the presence of a CVC or PICC during PIVC insertion, the catheter insertion site, and the total administered dose of nicardipine.
CI, confidence interval; CVC, central venous catheter; HR, hazard ratio; PICC, peripherally inserted central catheter; PIVC, peripheral intravenous catheter

**eFigure 6 Restricted cubic spline curve with three degrees of freedom using the standard Cox regression model (Model 4)**

The x-axis represents the total administered noradrenaline dose (mg), and the y-axis represents the HR for each total administered noradrenaline dose, with the reference being the total dose of noradrenaline set to zero. The solid red line indicates the HR estimated using multilevel standard Cox regression model, treating the total dose of noradrenaline as a time-independent factor, and the orange dashed line indicates the 95% CI (degrees of freedom: 3). The overall HR and 95% CI were 1.11 (1.04–1.20), and the linearity test result was p < 0.001. The covariates included in Model 4 were age, sex, body mass index, APACHE II score, insertion site, catheter gauge, CVC/PICC insertion, drug administration standardization, and total dose of amiodarone, ampicillin/sulbactam, cefepime, ceftriaxone, meropenem, nicardipine, potassium, PPN, propofol, and vancomycin.
APACHE, Acute Physiology and Chronic Health Evaluation; CI, confidence interval; CVC, central venous catheter; HR, hazard ratio; PICC, peripherally inserted central catheter; PIVC, peripheral intravenous catheter; PPN, peripheral parenteral nutrition
